# Molecular features and predictive models identify the most lethal subtype and a therapeutic target for osteosarcoma

**DOI:** 10.3389/fonc.2023.1111570

**Published:** 2023-02-16

**Authors:** Kun Zheng, Yushan Hou, Yiming Zhang, Fei Wang, Aihua Sun, Dong Yang

**Affiliations:** ^1^ State Key Laboratory of Proteomics, Beijing Proteome Research Center, National Center for Protein Sciences (Beijing), Beijing Institute of Lifeomics, Beijing, China; ^2^ Department of Orthopedics, General Hospital of Southern Theater Command, Guangzhou, China; ^3^ Research Unit of Proteomics Driven Cancer Precision Medicine, Chinese Academy of Medical Sciences, Beijing, China

**Keywords:** osteosarcoma, molecular classification, cholesterol metabolism, drug target, SQLE, predictive model

## Abstract

**Background:**

Osteosarcoma is the most common primary malignant bone tumor. The existing treatment regimens remained essentially unchanged over the past 30 years; hence the prognosis has plateaued at a poor level. Precise and personalized therapy is yet to be exploited.

**Methods:**

One discovery cohort (n=98) and two validation cohorts (n=53 & n=48) were collected from public data sources. We performed a non-negative matrix factorization (NMF) method on the discovery cohort to stratify osteosarcoma. Survival analysis and transcriptomic profiling characterized each subtype. Then, a drug target was screened based on subtypes’ features and hazard ratios. We also used specific siRNAs and added a cholesterol pathway inhibitor to osteosarcoma cell lines (U2OS and Saos-2) to verify the target. Moreover, PermFIT and ProMS, two support vector machine (SVM) tools, and the least absolute shrinkage and selection operator (LASSO) method, were employed to establish predictive models.

**Results:**

We herein divided osteosarcoma patients into four subtypes (S-I ~ S-IV). Patients of S- I were found probable to live longer. S-II was characterized by the highest immune infiltration. Cancer cells proliferated most in S-III. Notably, S-IV held the most unfavorable outcome and active cholesterol metabolism. SQLE, a rate-limiting enzyme for cholesterol biosynthesis, was identified as a potential drug target for S-IV patients. This finding was further validated in two external independent osteosarcoma cohorts. The function of SQLE to promote proliferation and migration was confirmed by cell phenotypic assays after the specific gene knockdown or addition of terbinafine, an inhibitor of SQLE. We further employed two machine learning tools based on SVM algorithms to develop a subtype diagnostic model and used the LASSO method to establish a 4-gene model for predicting prognosis. These two models were also verified in a validation cohort.

**Conclusion:**

The molecular classification enhanced our understanding of osteosarcoma; the novel predicting models served as robust prognostic biomarkers; the therapeutic target SQLE opened a new way for treatment. Our results served as valuable hints for future biological studies and clinical trials of osteosarcoma.

## 1 Introduction

Osteosarcoma is the most prevalent primary malignant tumor of bone (1) and has an annual incidence of 2-3 cases per million individuals (2), accounting for 0.2% of human malignancies and 11.7% of primary bone tumors (3–6). It most frequently occurs in adolescents (4.4 cases per million individuals) (7), which coincides with the growth spurt. The second peak of the incidence occurs in adults aged > 65 years (4.2 cases per million individuals) (7, 8). Besides, 80%~90% of osteosarcoma is diagnosed in long tubular bones, with the most common sites being the distal femur and proximal tibia, followed by the proximal humerus (9). Moreover, about 85% of metastases tend to disseminate to the lungs, and most cases occur within two years (10). However, the exact etiology and pathogenesis of osteosarcoma remain unclear.

Due to highly aggressive malignancy, osteosarcoma patients persistently endure severe pain and an increased risk of pathological fracture (11, 12). Regardless of whether the tumor is localized or metastatic, patients have to undergo standard treatment regimens consisting of rigorous multidrug therapy and extensive surgical resection (13). Despite continuous attempts to refine the established treatment protocol, improvements in survival outcomes for osteosarcoma have plateaued over the past 30 years (14). Osteosarcoma being metastatic or recurrent portends a bleak prognosis with a 5-year survival rate below 30% (15). Additionally, osteosarcoma cases are prone to be resistant to intensive chemotherapy, leading to severe toxicities and increased morbidities in neutropenia, infective complications, and thrombocytopenia (16).

In recent years, a growing number of studies utilizing whole genome sequencing (WGS) or single-cell RNA landscape led to a deeper understanding of osteosarcoma’s genome features and immunological signatures (17). For example, osteosarcoma is distinguished by plenty of copy number variations (CNVs) with few single-nucleotide variations (SNVs) except for TP53 and RB1 (15). These mutations remain non-applicable for targeted therapy (18). Clinical trials of targeted therapy are currently being conducted around the world; however, no effective drug has been proven available for osteosarcoma until now (19). Thus, it is urgent to develop novel strategies for addressing the current treatment dilemma of osteosarcoma.

Omics data mining and machine learning models have become increasingly popular in stratifying patients and further exploring biological information hidden behind the high-throughput sequencing data of osteosarcoma and other solid tumors (20, 21). In 2020, Chia-Chin Wu, et al. (17) conducted multi-omics sequencing and used single sample gene set enrichment analysis (ssGSEA) to identify osteosarcoma with three clusters based on levels of immune infiltrate. Their data and classification reveal multiple immunosuppressive features of osteosarcoma. In 2021, Song, Y.J., et al. (22) stratified osteosarcoma into two subtypes according to the tumor microenvironment (TME) and described the immunological features of its subtypes. Deyao Shi, et al. (23) used DNA methylation profiles to categorize patients into three subgroups. They found distinct prognoses and tumor microenvironment patterns across subgroups. All these osteosarcoma stratifications mentioned above were mainly focused on the divergence of immune infiltration. However, immune checkpoint inhibitors (ICI) treatment, including the PD-1/PD-L1 treatment, which contributes to a breakthrough in immunotherapy for many solid tumors, has limited therapeutic effects in osteosarcoma so far (24). Up to now, no currently available classifier can comprehensively delineate the metabolic features of each subtype, which severely limits the ability of researchers to explore more individualized treatments for this disease.

To refine the current treatment regimen with more personalized therapeutic options, we herein divided osteosarcoma patients into four subtypes and provided a full view of prognostic differences and enriched pathways. Then our study placed emphasis on subtype IV, which was characterized by a poor outcome and active cholesterol biosynthesis. Unprecedentedly, we proposed SQLE, a rate-limiting enzyme for cholesterol biosynthesis, as a potential drug target for S-IV patients. Knocking down SQLE or adding the inhibitors could suppress the proliferation and migration of osteosarcoma cells. Besides, we applied the support vector machine (SVM) algorithm to develop a subtype diagnostic model and used the least absolute shrinkage and selection operator (LASSO) method to establish a 4-gene model for identifying unfavorable outcomes. Our work served as a helpful reference for further improving efficacy in precision medicine of osteosarcoma.

## 2 Materials and methods

### 2.1 Study design

**Figure 1:**
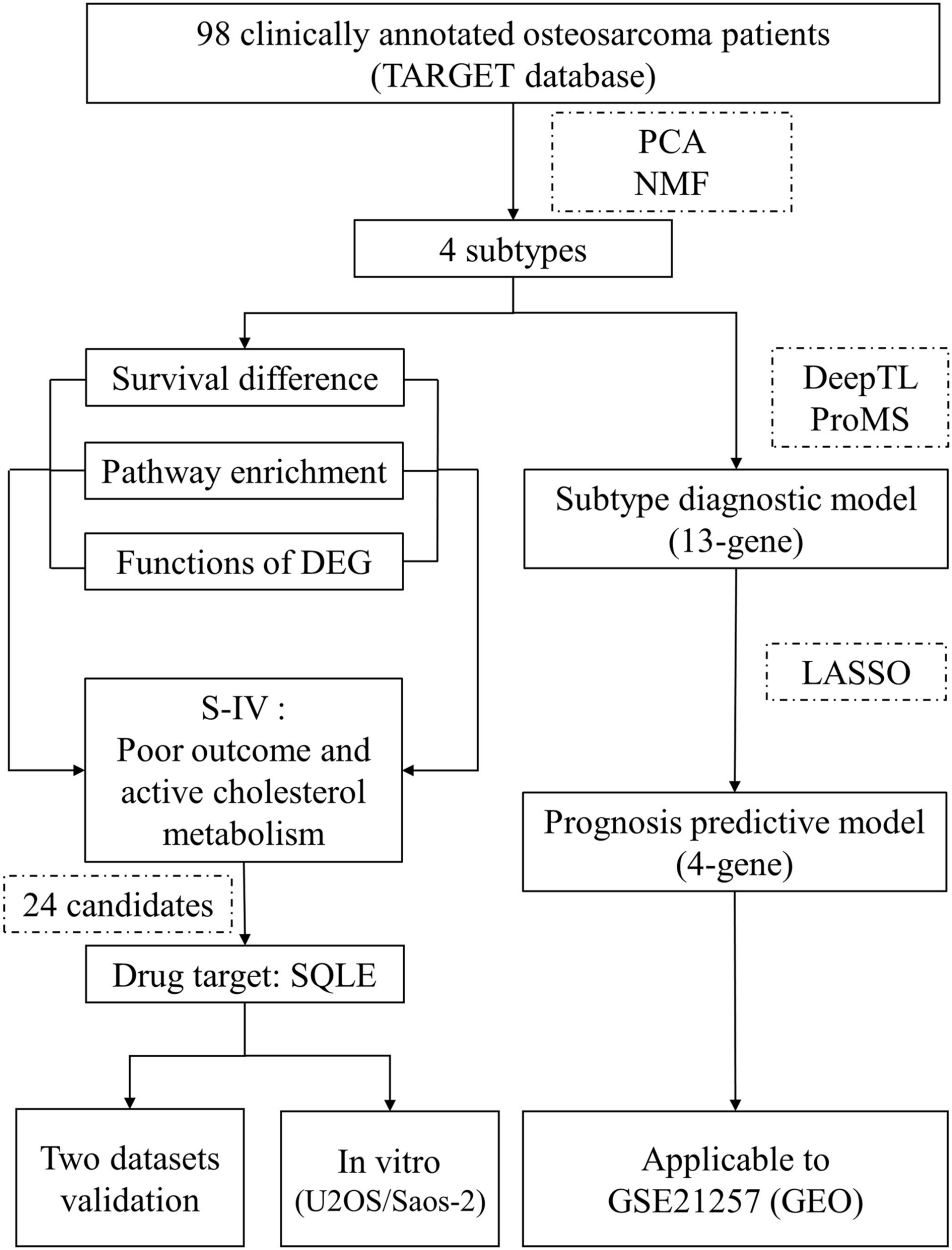
Outline of the study design. A dataset containing gene expression data from 98 osteosarcoma patients and relevant clinical information was utilized for molecular classification and subtype characterization. Features of subtype IV were mined to identify a drug target that fulfilled the “targeting cholesterol biosynthesis for living longer” implication. The screening yielded 24 candidates, of which only SQLE was encoded for a therapeutically actionable option. This drug target was tested in two independent cohorts and *in vitro* experiments. Moreover, we employed two machine learning tools to develop a 13-gene model to determine the subtype of osteosarcoma patients. Based on the 13 curated genes, we used the LASSO method to establish a 4-gene model to recognize patients with a significant unfavored outcome. These two models were both well evaluated by an independent dataset. TARGET, Therapeutically Applicable Research to Generate Effective Treatments; PCA, principal component analysis; NMF, non-negative matrix factorization; DEG, differentially expressed genes; S-IV, subtype IV; LASSO, least absolute shrinkage and selection operator; GEO, Gene Expression Omnibus.

Our overall study design is illustrated in **Figure 1**.

### 2.2 Patient population acquisition

We collected a training cohort coupled with two validation cohorts. All datasets are available from public data sources.

The training cohort comprised 98 clinically annotated patient cases produced by the Therapeutically Applicable Research to Generate Effective Treatments (TARGET) Osteosarcoma project (https://ocg.cancer.gov/programs/target/projects/osteosarcoma). Transcripts per million (TPM) values and read counts were downloaded from the TARGET data matrix. Furthermore, TPM values were scaled with Min-Max normalization.

The dataset GSE21257 containing clinical information of 53 osteosarcoma patients (25), was obtained from the Gene Expression Omnibus (http://www.ncbi.nlm.nih.gov/geo/). It was treated as a validation cohort to test the molecular subtyping and drug targets. The other validation dataset provided by the European Genome-phenome Archive (EGA, https://ega-archive.org) contains RNA-seq data from 48 specimens of osteosarcoma patients labeled with primary, relapsed, or metastatic (17). The accession number is EGAS00001003887.

### 2.3 NMF clustering and principal component analysis

The gene expression matrix of the 98 osteosarcoma samples was used to identify the molecular subtypes using the non-negative matrix factorization (NMF) consensus cluster method (26). NMF is a practical machine-learning approach for identifying molecular classification and has been widely used in many subtyping cases (27). Firstly, we filtered the genes expressed (read counts≥10) in less than 24 samples, and 26824 genes remained. Secondly, after min-max normalization, all TPM data were rescaled from 0 to 1. Thirdly, we performed principal component analysis (PCA) to select the top 1600 genes based on the sum of PC1 and PC2 rotation values. These 1600 genes and expression profiles were further subjected to NMF v.0.22.0 in R for unsupervised consensus-clustering. Non-smooth NMF (nsNMF) algorithm was employed with 100 iterations for the rank survey between 2 and 6 clusters.

### 2.4 Survival rate comparison among subtypes

The R package Survminer plotted the Kaplan-Meier survival curves. This tool employed a log-rank test to compare subtypes’ overall and event-free survival rates.

### 2.5 Tumor-infiltrating immune cell abundance calculation

Using the single sample gene set enrichment analysis (ssGSEA) method, xCell and ESTIMATE calculated the tumor-infiltrating immune cell content of cancer samples. xCell could compute the abundance of 64 different cell types and scores of immune, stroma, and microenvironment (28). ESTIMATE could take advantage of the unique properties of the transcriptional profiles of cancer samples to infer tumor cellularity as well as the different infiltrating normal cells (29). They were two efficient publicly available algorithmic tools.

### 2.6 Differentially expressed genes identification

The differentially expressed genes (DEGs) were identified as highly expressed in one subtype compared to all three other subtypes. The limma package in R performed this computation. Adjusted P-values were applied for multiple testing corrections through the default Benjamini-Hochberg false discovery rate (FDR) method (30). Adjusted P-value < 0.05 and |fold change (FC)| > 1.5 were set as the cutoff values to determine DEGs.

### 2.7 Functional enrichment analyses

Gene set enrichment analysis (GSEA) was performed on all genes. GSEA was run with Molecular Signatures Database (MSigDB) set V7.4, C2: curated gene sets (browse 6290 gene sets) (31). In addition, enrichment analyses of gene ontology (GO), Reactome, and Kyoto Encyclopedia of Genes and Genomes (KEGG) pathway, were conducted on the DEG by using the R package clusterProfiler (version 4.2.2) (32).

### 2.8 PPI network analysis

The PPI (protein-protein interactions) network was constructed *via* an open-source platform Cytoscape (33). Using the Search Tool for the Retrieval of Interacting Genes/Proteins (STRING) database (https://string-db.org), the PPI network illustrated how proteins interrelate functionally and physically with each other by encoding the gene list as input (34).

### 2.9 Candidate drug targets identification

Druggable genes for osteosarcoma were mined using the Drug–Gene Interaction Database (DGIdb 4.0, https://www.dgidb.org). This database contained drug targets, indications, MOA, and drug status from over thirty reputable sources, including DrugBank, FDA, and NCI (35). The hazard ratios (HR) were computed with log-rank tests using univariate analysis based on a cox proportional hazards regression model. In addition, we used the Survminer R package to calculate the log-rank p values and the optimal cut-off values to stratify patients into high/low gene expression groups and further visualize the survival differences between groups. High-risk genes were identified with HR>1 and log-rank p<0.05.

### 2.10 Cell culture

The U2OS and Saos-2, human osteosarcoma cell lines, were obtained from the American Type Culture Collection (ATCC, VA, USA). The U2OS was cultured in high glucose DMEM supplemented with 10% fetal bovine serum (FBS), and the Saos-2 was cultured in McCoy’s 5A supplemented with 15% FBS. They were incubated at 37°C in 5% CO_2._ 1% penicillin and streptomycin (Gibco, USA) were added to the base media as supplements.

### 2.11 Gene silencing

Knockdown of SQLE in cells was achieved by using Turbofect transfection reagent (Thermo Fisher Scientific, OR, USA). 2 μL Turbofect reagent was mixed with 500 μL Opti-MEM (Thermo Fisher Scientific) and combined with 1 μg siRNA (GenePharma, Shanghai, China). The mixture was incubated at room temperature for 20 minutes and added dropwise to the cells. The targeted oligos were as follows (5’-3’):

siSQLE#1: GCCUCUAAAUCUUUAGGUUTT;
siSQLE#2: GCCCAGGUUGUAAAUGGUUTT;
siSQLE#3: GCUCAGGCUCUUUAUGAAUTT;

siNegative control: UUCUCCGAACGUGUCACGUTT.

### 2.12 Real−time quantitative PCR

Total RNA was extracted by Trizol reagents (Thermo Fisher Scientific). After reverse transcription by HiScript III All-in-one RT SuperMix (Vazyme, Nanjing, China), the mRNA levels were measured by SYBR master mix dye (Vazyme). The relative gene expression level was analyzed using the 2-ΔΔ Ct method, and GAPDH was used as the internal reference gene. The Q-PCR primer sequences were as follows (5’-3’): SQLE, forward, GGCATTGCCACTTTCACCTAT; reverse, GGCCTGAGAGAATATCCGAGAAG. GAPDH, forward, TGCACCACCAACTGCTTAGC; reverse, GGCATGGACTGTGGTCATGAG. QPCR was conducted after 24h siRNA transfection.

### 2.13 Western blot analysis

Total protein was extracted by RIPA lysis buffer (Thermo Fisher Scientific). After BCA quantification, equal volumes of protein samples were separated by 10% SDS-PAGE and transferred to a 0.45 nitrocellulose filter membrane (Millipore, MA, USA). The immunoblots were sequentially probed with the SQLE primary antibody (Cat No. 12544-1-AP, Proteintech, Wuhan, China) and secondary antibody (Cat No. SA00001-2, Wuhan, China). Finally, the detection was performed using an ECL chemical luminescent detection kit (Thermo Fisher Scientific), and the bands were further analyzed using ImageJ software. The levels of the target protein were normalized to β-actin expression. Western blot analyses were conducted after 48h siRNA transfection.

### 2.14 CCK8 assays

Terbinafine was purchased from Selleckchem (Houston, TX, USA). Cells were plated in 96-well plates at a density of 3,000 cells; the indicated siRNAs or terbinafine were introduced to cells after 12 h. When it came to the endpoint of the assay, 10% CCK8 reagent (Dojindo, Kyushu Island, Japan) was added into the wells; after 2 h incubation at 37°C, the OD values were detected at 450 nm.

### 2.15 Colony formation assays

Cells treated by siRNAs or terbinafine were plated in 6-well plates at a density of 2,000 cells and then cultured for two weeks. The cells were fixed and stained with 0.1% crystal violet (Beyotime, Beijing, China).

### 2.16 Transwell assays

15,000 cells were plated in the upper chamber for the silencing cells, while DEME containing 10% FBS was added to the lower chambers. For the inhibitor-treated cells, cells in the upper chamber were treated with terbinafine (25 μM, 50 μM) for 24h in advance, while DEME containing 10% FBS was added to the lower chambers. After 24h incubation, the crossed cells were fixed and stained with 0.1% crystal violet.

### 2.17 Intracellular cholesterol assays

Treated cells were washed with PBS twice and resuspended in isopropanol containing 1% Triton X-100 for 30 min at room temperature. After centrifugation at 12,000 rpm for 15 min, the supernatants were dried under nitrogen. The powders were dissolved with assay buffers. Cholesterol measurements were performed according to the instruction guide of the Amplex™ Red cholesterol assay kit (Thermo Fisher Scientific).

### 2.18 Machine learning models for diagnosis and prognosis prediction

The subtype diagnostic model was constructed using a supervised learning method SVM. Permutation-based feature importance test (PermFIT) implemented in the R package “deepTL” (https://github.com/SkadiEye/deepTL) was proposed for assisting the interpretation of individual features in complex frameworks including SVM (36). Herein, we conducted PermFIT-SVM on expression profiles of DEG with a threshold of p-value<0.05 and thereby obtained 16, 25, 32, and 69 feature genes for S-I, S-II, S-III, and S-IV subtypes, respectively.

ProMS (https://github.com/bzhanglab/proms) is a unified and effective computational framework containing an SVM classifier for feature selection with the help of an omics view (*e.g.*, RNA-seq) (37). We applied ProMS-SVM on feature genes of each subtype for training and testing the subtype diagnostic model. Preliminary runs were repeated 20 times, from K=1 to K=5, with the percentiles of fifth, 10th, 15th, 20th, and 25th. Then, runs were repeated 100 times to confirm the model’s accuracy. Hyperparameters were tuned using grid search with 3-fold cross-validation to select the best model *via* measuring the area under the receiver operating curve (AUC) value.

### 2.19 LASSO logistic regression modeling

Utilizing patient information and expression data of 13 genes from SDM, the model’s genes were rigorously screened out using the R package “glmnet” (38) to repeat 5-fold cross-validation 100 times. The optima λ (λ.min) was selected to minimize the penalization in each iteration of the LASSO regression analysis. The number of model genes was determined by the most frequent gene numbers when setting λ.min and coefficient ≠ 0. Then, genes with the most frequencies were retained and combined as significant predictors of mortality. Finally, another 100 iterations were conducted to determine the coefficient of each signature gene.

## 3 Results

### 3.1 Molecular classification of osteosarcoma and characteristics of each subtype

**Figure 2:**
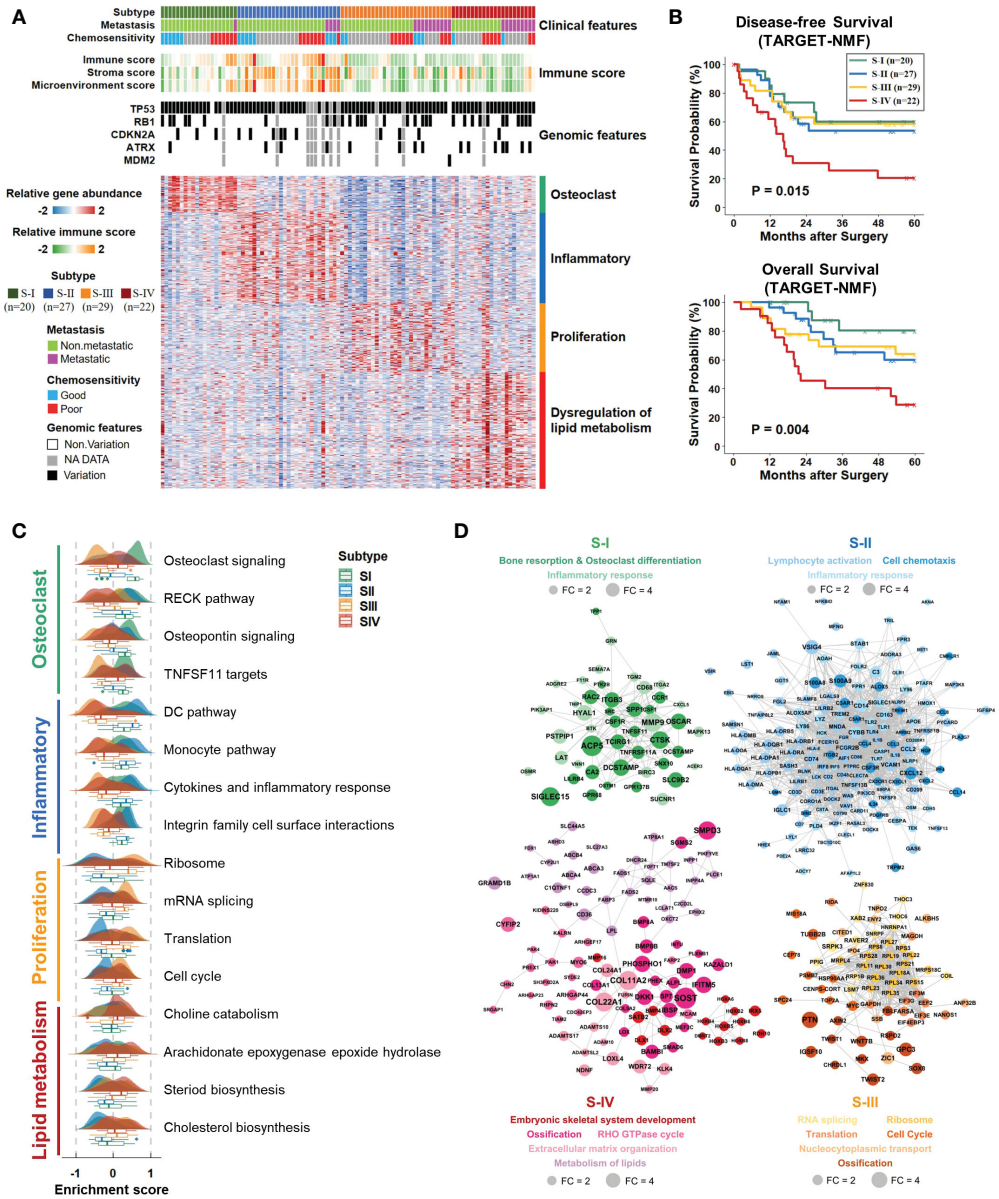
Clinicopathologic correlations and functional features of each subtype. **(A)** Consensus-clustering analysis of transcriptomic profiling identified four subtypes (Tumor samples, n = 98): S-I (green, n = 20), S-II (blue, n = 27), S-III (yellow, n = 29) and S-IV (red, n=22). Each column indicated a patient sample, and rows represented genes. The associations of subtypes with clinical, immune, and genomic characteristics were annotated in the middle panel. The heat map below depicted the relative abundance of signature genes (log2-transformed). Biological functions were denoted on the right, based on the enrichment of functional pathways. **(B)** Kaplan–Meier curves of disease-free and overall survival for each subtype in the TARGET cohort. P values were calculated by log-rank test. **(C)** Ridge and box plots depicted the enrichment of representative GSEA biological pathways in each subtype. **(D)** PPI network described functional enrichment analyses of DEG (FC>1.5, adjusted *p* value<0.05). Each dot corresponded to a gene. The dot color represented the subtype, the size fit the fold-change, and different transparency referred to different functional categories. FC, fold change; GSEA, gene set enrichment analysis; PPI, protein-protein interactions.

The NMF algorithm, an effective consensus-clustering method, was employed for osteosarcoma subtype identification. The cophenetic score and silhouette width of the rank survey profiles indicated a potential option of four-subtype (**Supplementary Figures 1A, B**). Furthermore, we conducted 400 iterations for the clustering runs in the case of rank=4. The consensus clustering heat maps (**Supplementary Figure 2**) illustrated that four-subtype is an appropriate solution for osteosarcoma (**Figure 2A**; **Table S1**). Hereinafter referred to as S-I, S-II, S-III, and S-IV. The TARGET database provided clinical information and genomic alteration (**Table S2**). According to immune scores computed by xCell (**Table S3**) and expression level of immune-related genes (**Supplementary Figures 3A, B**), S-II had the highest tumor-infiltrating immune cell abundance. In addition, overall survival and disease-free survival varied among subtypes (**Figure 2B**). Patients of S-IV showed the most unfavorable outcome.

To obtain the biological signatures of each subtype, we performed functional enrichment analyses on all genes and DEG (**Tables S4**, **S5**). GSEA conducted on all genes (**Figure 2C**) demonstrated that patients of S-I tended to exhibit more osteoclastic bone resorption (e.g., ACP5, SIGLEC15, CTSK), which was consistent with the clinical manifestations of osteosarcoma at the early stage (39). Multiple immune pathways were enriched in S-II (e.g., IGKC, S100A9, CD14), while S-III was prone to manifest salient cell proliferation features (e.g., TUBB2B, MYC). Patients of S-IV showed markedly enhanced lipid metabolism (e.g., SQLE, CD36, LPL), including cholesterol biosynthesis, which was believed to be a critical driven factor in the development of some solid tumors (40, 41). Apart from GSEA analysis, functional enrichment of DEG also revealed that S-I mainly correlated with bone resorption and osteoclast differentiation. Remarkable enrichment in immune-related functions was found in S-II. Various processes involved primarily in cancer cell proliferation were exhibited in S-III. Several functional categories, including the metabolism of lipids, were enriched in S-IV (**Figure 2D**).

### 3.2 Cholesterol biosynthesis was found particularly active in S-IV and probably led to a poor prognosis

**Figure 3:**
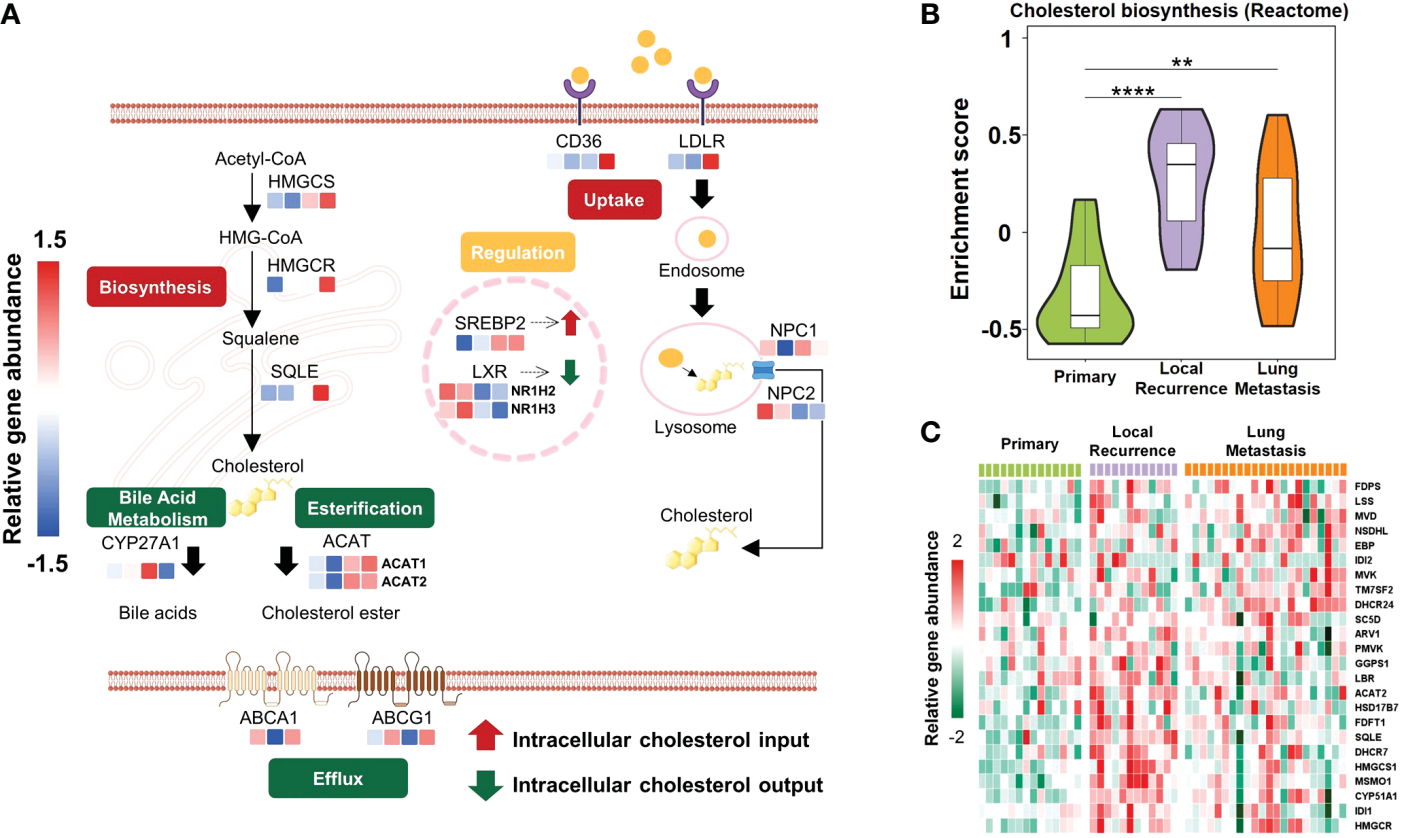
Schematic diagram showing active cholesterol metabolism in patients with unfavorable outcomes. **(A)** Mechanisms regulating cholesterol homeostasis in different subtypes, including repletion (biosynthesis, uptake) and depletion (esterification, bile acid metabolism, efflux). *De novo* biosynthesis pathway started from acetyl-CoA and HMG-CoA and stepwise provided squalene for cholesterol synthesis. This process was mediated by rate-limiting enzymes HMGCR and squalene epoxidase (SQLE). Besides biosynthesis, cells also acquire cholesterol from low-density lipoprotein (LDL) uptake *via* LDLR-mediated endocytosis. Excessive cholesterol was utilized as a precursor to generating bile acids by CYP27A1 or was esterified by acyl-coenzyme A: cholesterol acyltransferases (ACATs; also known as SOATs). Furthermore, cholesterol efflux was regulated by ATP-binding cassette (ABC) transporters such as ABCA1 and ABCG1. In the nucleus, sterol regulatory element binding protein 2 (SREBP2) and liver X receptor (LXR) were two transcription factors that reciprocally regulated cholesterol metabolism. Colored rectangles from left to right represent S-I, S-II, S-III, and S-IV subtypes in the same row. The color of each rectangle showed *Z*-score (log_2_ of relative abundance scaled by genes’ SD) of the gene in that sample; red showed high expression while blue showed low. **(B)** Violin plots exhibited the various enrichment of cholesterol biosynthesis pathways between relapsed or metastatic lesions and primary lesions. The width of the violin plot represented the number of samples at the given enrichment score on the height. Data were derived from the cohort from European Genome-phenome Archive (EGA cohort). **P<0.01, ****P<0.0001 by Wilcoxon-test. **(C)** Heatmap depicted proteins involved in cholesterol biosynthesis pathways (rows) with the expression level of that sample type in the EGA cohort (columns). Red indicated the high expression; green showed the low.

Cholesterol metabolism produces metabolites with various biological functions and plays complex roles in tumorigenesis (42). Clinical trials manipulating cholesterol metabolism to inhibit tumorigenesis and reinvigorate anti-tumor immunity are ongoing (43). Overall, cholesterol homeostasis is maintained by the balance of *de novo* biosynthesis, cholesterol uptake, bile acid metabolism, esterification, and efflux (44). To decipher whether cholesterol metabolism was reprogrammed in patients of different osteosarcoma subtypes, we identified expression features of critical regulators that contribute to various modules (**Figure 3A**; **Table S6**). Cholesterol accumulation was facilitated by cholesterol biosynthesis and cellular uptake of LDL-cholesterol, while cholesterol consumption was mainly regulated by cholesterol esterification and efflux (45). When cholesterol accumulates to a high level, cells relieve cellular stress through esterification and efflux (46). In S-IV patients, mediators involved in cholesterol biosynthesis (HMGCS, HMGCR, SQLE) and cholesterol consumption (ACAT1, ACAT2, ABCA1, ABCG1) were highly expressed, while regulators promoting cellular uptake of LDL-cholesterol (LDLR, Niemann-Pick C proteins including NPC1 and NPC2) were not active. Especially, NPC2 was significantly depleted in S-IV. Besides, massive intracellular cholesterol limited the translocation of the SREBP2 to the nucleus, reducing its transcriptional target LDLR (47). The LDLR depletion suppressed the cholesterol uptake pathway and its downstream regulators NPC1/NPC2. These findings suggested cholesterol biosynthesis was particularly active in S-IV, leading to cholesterol accumulation. As a result of negative feedback regulation, cellular uptake of cholesterol was inhibited.

In addition to the TARGET cohort, GSEA performed on the EGA cohort (**Table S7**) indicated that the cholesterol biosynthesis pathway was more enriched in recurrent or pulmonary metastases than in primary lesions (**Figure 3B**). Furthermore, according to gene expression level comparison, almost all the genes involved in this pathway were observably highly expressed in relapsed or metastatic tumors (**Figure 3C**; **Table S8**). Since recurrence or metastasis lead to treatment failure in most cases (48), and S-IV patients were predicted with poor prognosis, a hint was uncovered that active cholesterol biosynthesis could probably confer an unfavorable outcome.

### 3.3 SQLE was identified as a potential drug target for osteosarcoma patients of S-IV

As patients of S-IV tended to exhibit active cholesterol biosynthesis with a poor prognosis, and this pathway was strongly associated with a poor prognosis in the EGA cohort, we speculated that S-IV patients could benefit from targeting the cholesterol biosynthesis process.

**Figure 4:**
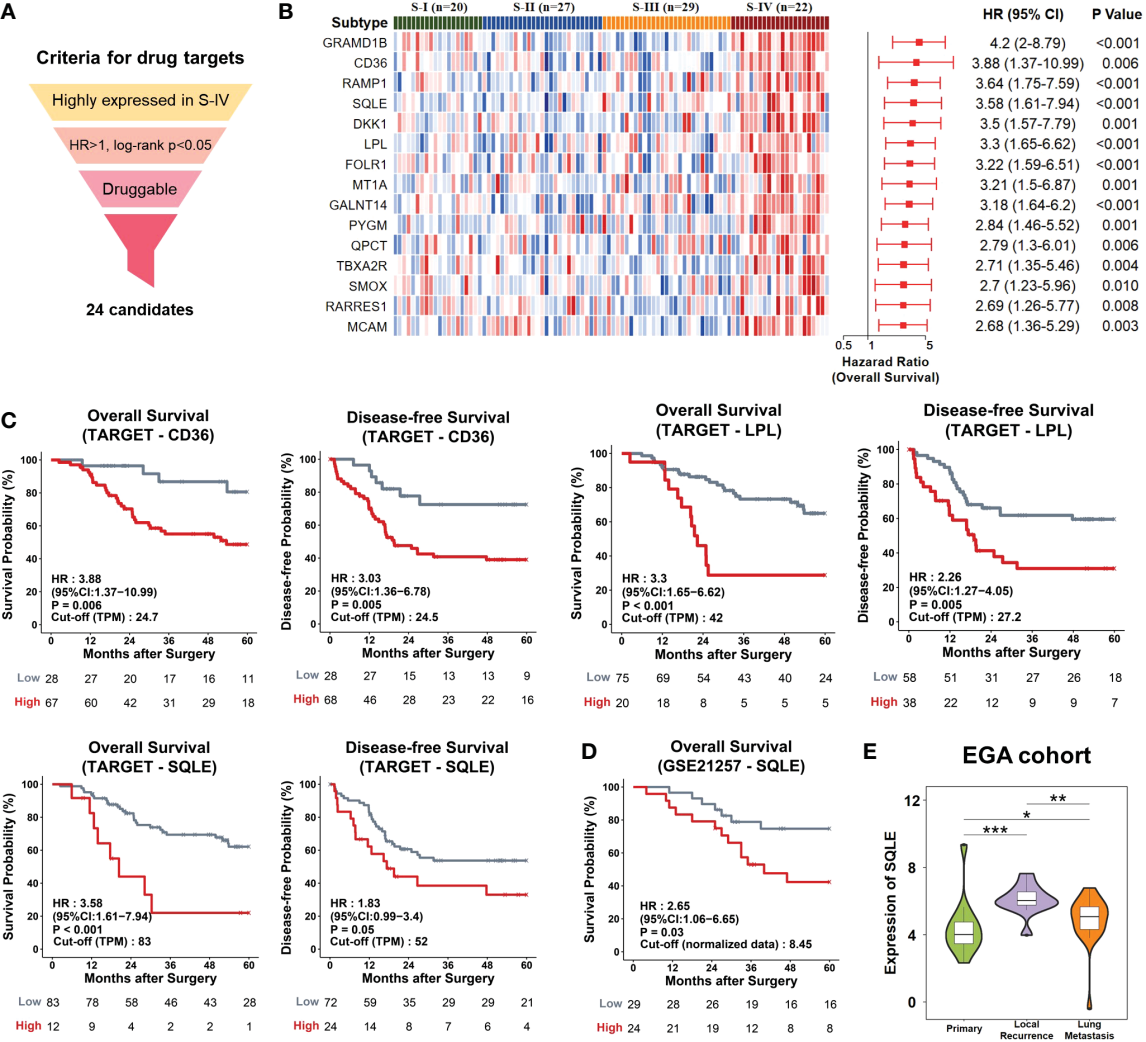
Potential drug targets for osteosarcoma patients. **(A)** The workflow of the drug-target screening pipeline. We set three layers of filtration and obtained 24 candidates. **(B)** The candidates were ranked by hazard ratio (HR), and the top 15 were chosen to be presented. Left, Heatmap depicted the relative abundances of the top 15 candidates. Right, Forrest plot showed risk scores of genes. The red points denoted the HR of overall survival. The endpoints indicated lower or upper of the 95% confidence intervals (CI). P-values were calculated by log-rank test. **(C)** Kaplan-Meier curves. The TARGET patients were divided into high/low gene expression groups using the optimal cut-off value. Patients with high expression levels of CD36, LPL, or SQLE displayed poor outcomes. **(D)** The GSE21257 patients with high expression levels of SQLE displayed poor outcomes. **(E)** Violin plots showed the distributions of expression levels of SQLE in the EGA samples with different pathological stages. The width represented the number of samples at the given expression level on the height. *P<0.05, **P<0.01, ***P<0.001 by Wilcoxon-test.

Genes highly expressed in S-IV, showing high-risk scores for mortality (HR>1, log-rank p<0.05), and druggable in the DGIdb database, were identified as potential candidates for targeted therapy. 24 candidates fit the criteria above (**Figure 4A**; **Table S9**). They were ordered by decreasing HR values and top 15 were presented (**Figure 4B**). Among these candidates, CD36, SQLE, and LPL participated in cholesterol metabolism. Each over-representation led to an apparent inferior overall and disease survival (**Figure 4C**). SQLE is one of the rate-limiting enzymes in cholesterol biosynthesis (49). Meanwhile, Food and Drug Administration (FDA)-approved drugs targeting SQLE have been used in trials exploring the treatment of various cancers (50, 51). The impacts of SQLE on the prognosis of the GSE21257 dataset and the EGA cohort were also examined; high expression portended a poor prognosis (**Figure 4D**) and a significant trend of recurrence or metastasis (**Figure 4E**). Hence, we proposed that SQLE was a suitable drug target for osteosarcoma patients of S-IV.

### 3.4 Cellular phenotypic experiments well verified the potential drug target SQLE

**Figure 5:**
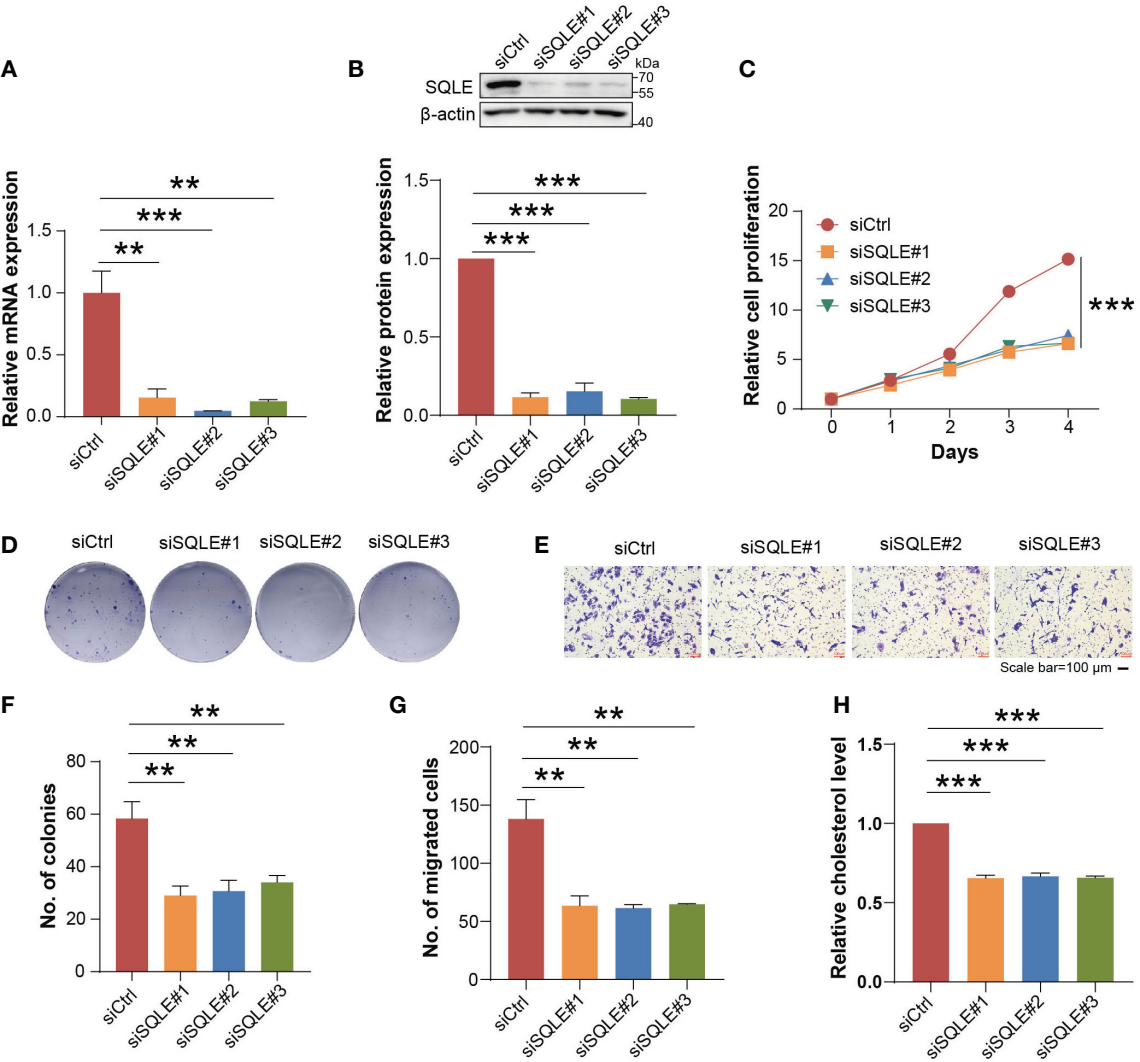
SQLE knockdown produced significant inhibition on cancer-related phenotypes of U2OS cells *in vitro*. **(A)** Quantitative PCR (Q−PCR) with mRNA expression confirmed the knockdown of SQLE after 24h siRNA transfection. **(B)** Western blot analyses with protein expression confirmed the knockdown of SQLE after 48h siRNA transfection. The β-actin was treated as the loading control. **(C)** Cell proliferation was suppressed by SQLE knockdown. **(D)** Cell colony formation ability was reduced by SQLE knockdown. **(E)** Cell migration was inhibited by SQLE knockdown. The scale bar represented 100 μm. **(F)** and **(G)** indicated the quantification of positive signals from **(D, E)**, respectively. **(H)** The knockdown of SQLE significantly reduced the intracellular cholesterol. Error bars represented ± SD of three biological replicates. **P < 0.01, ***P < 0.001 by Student t-test.

**Figure 6:**
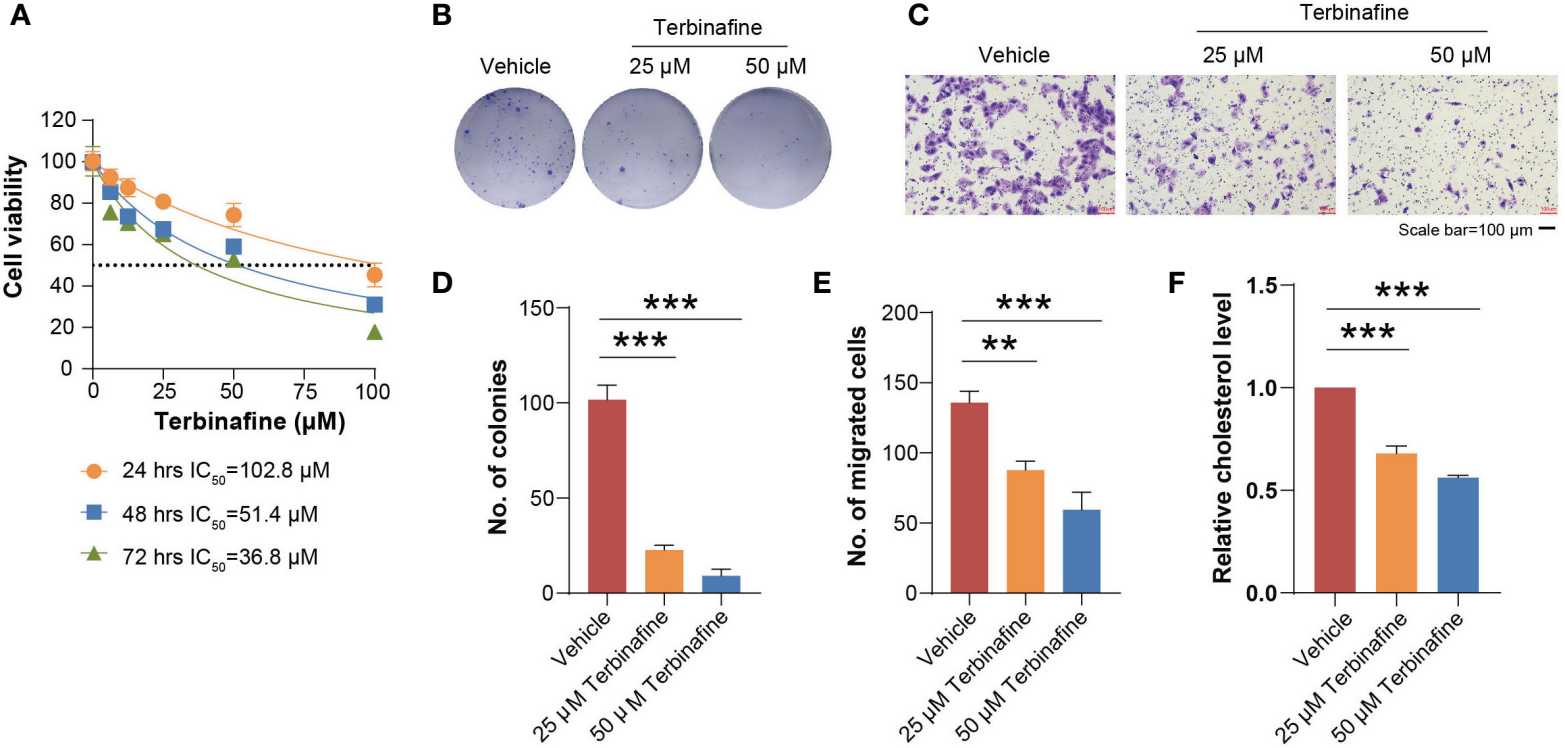
Terbinafine treatment impaired the growth and migration of U2OS cells *in vitro*. **(A)** Different gradient concentrations of terbinafine were added to the U2OS cells. As the dosage of terbinafine increased, the viability of the cells decreased. **(B)** The U2OS cells were respectively treated with 25 μM and 50 μM terbinafine. Cell colony formation ability was found reduced with the increasing dosage of terbinafine. **(C)** The number of migrating cells decreased obviously with the increase in drug concentration. Scale bars, 100 μm. **(D, E)** indicated the quantification of positive signals from **(B, C)**, respectively. **(F)** The terbinafine treatment significantly reduced the intracellular cholesterol. Error bars represent mean ± SD; statistical analysis was performed using the Student t-test. **P < 0.01, ***P < 0.001.

To evaluate the contribution of SQLE to the phenotype associated with tumor growth, we knocked down SQLE *via* specific siRNAs in osteosarcoma cells, including U2OS and Saos-2 cell lines. To confirm the knockdown effect of SQLE, Q-PCR and western blot were used to detect SQLE expression at the mRNA and protein levels, respectively (**Figures 5A, B**; **Supplementary Figures 4A, B**). Experimental results indicated that the silencing of SQLE could substantially inhibit cell proliferation, colony formation, and migration (**Figures 5C–G**, **Supplementary Figures 4C–E**). Furthermore, we examined the effect of terbinafine, a specific inhibitor of SQLE, on the cell phenotype. According to a conventional pharmacodynamic measure IC50 (drug concentration causing 50% inhibition), terbinafine was found to inhibit cell viability in a time- and dose-dependent manner (**Figure 6A** and **Supplementary Figure 5A**). Next, we set the concentration gradient of terbinafine based on the value of IC50 at 48h. The colony formation ability decreased dose-dependently (**Figures 6B, C**), and the migrated cells were reduced with the gradient of terbinafine challenge (**Figures 6D, E**; **Supplementary Figures 5B, C**). Meanwhile, both SQLE knockdown and terbinafine treatment decreased intracellular cholesterol (**Figure 5H**; **Supplementary Figure 4F**, **Figure 6F**, and **Supplementary Figure 5D**). Due to the negative feedback regulation of intracellular cholesterol, the protein abundance of SQLE was dose-dependently enhanced by terbinafine challenge in U2OS and Saos-2 cells. (**Supplementary Figures 6A, B**). These results signified that targeting SQLE could reduce proliferating and migrating ability of osteosarcoma *via* reducing intracellular cholesterol. Thus, SQLE could be a potential therapeutic target for osteosarcoma patients.

### 3.5 Subtype diagnostic model and LASSO model

**Figure 7:**
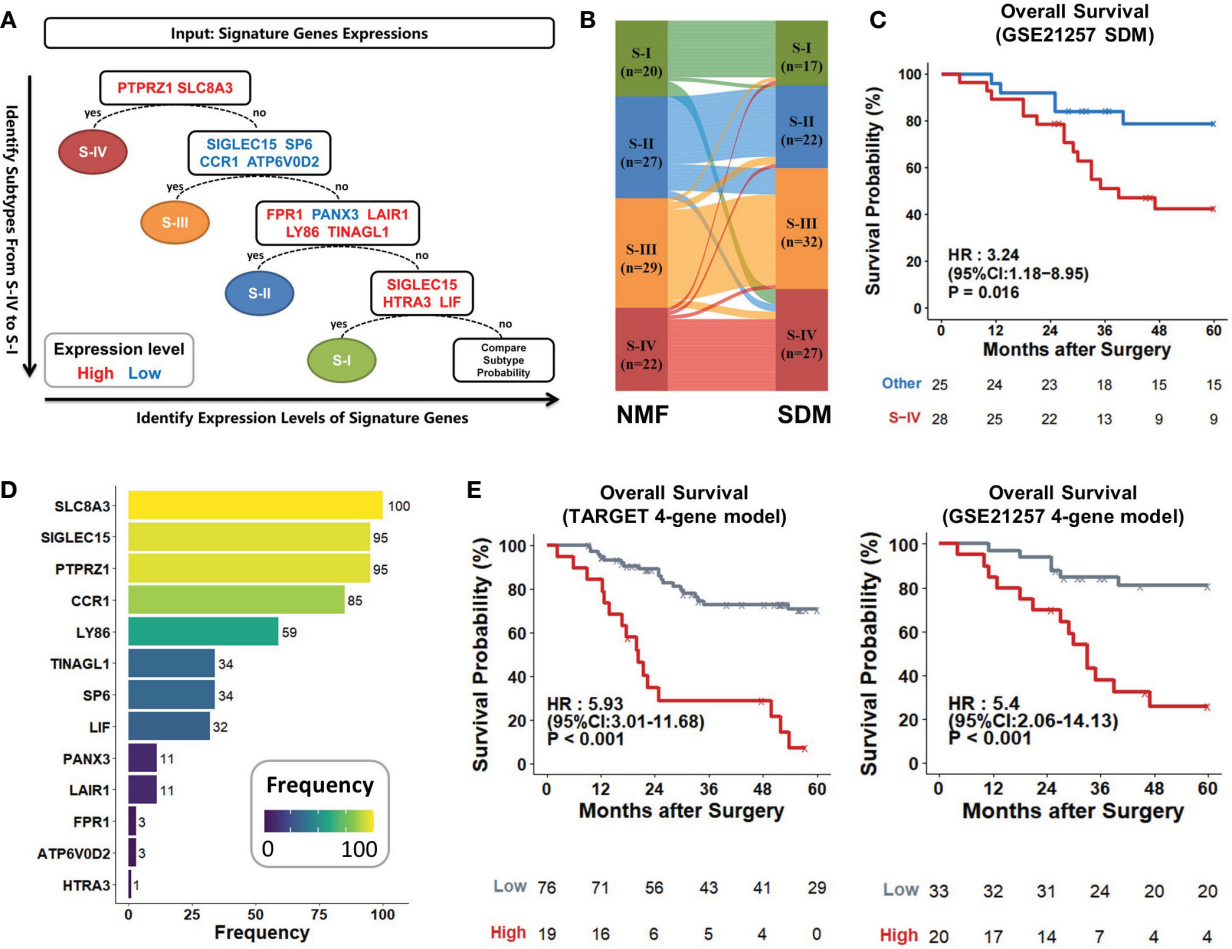
Establishment and evaluation of two predictive models. **(A)** Schematic workflow of the subtype diagnostic model. Osteosarcoma patients were sequentially stratified according to 13 marker genes with high (red) or low (blue) expressions. **(B)** Sankey diagram showed that subtypes identified by SDM matched very closely with those by the NMF method. Each rectangle represented a subtype, and each row represented a patient sample. The connection degree was visualized based on the size of rectangles. **(C)** Kaplan–Meier curves of S-IV and other patients predicted by SDM in the GSE21257 dataset (S-IV, n = 28; others, n = 25). **(D)** PTPRZ1, SLC8A3, SIGLEC15, and CCR1 were the top four genes that were selected most frequently within the 100 iterations. They were retained to develop the LASSO model. **(E)** Kaplan-Meier curves. The 4-gene model with high scores led to poorer outcomes compared to the low in the TARGET cohort and GSE21257 dataset. *P* values were calculated by log-rank test.

Identifying subtypes of osteosarcoma patients at initial biopsy could help make more precise therapeutic strategies early and optimize treatment efficacy (52). We used the PermFIT to determine 142 feature genes for subtypes (**Table S10**) and employed the machine learning approach SVM by ProMS to build the subtype diagnostic model (SDM). This model was composed of 13 genes with high or low expression levels (**Table S11**). Patients were subtyped in sequence of S-IV, S-III, S-II, and S-I (**Figure 7A**). To assess the accuracy of the SDM, we applied this model to distinguish the discovery cohort. Most patients were grouped into identical subtypes in this model compared with the NMF approach (**Figure 7B**; **Table S12**). We also applied SDM to the GSE21257 dataset; survival curves suggested that S-IV generated poorer outcomes than the rest (**Figure 7C**; **Table S13**). These results reflected that our SDM achieved excellent prediction accuracy.

To recognize grim prognosis using fewer genes, we established a prognostic classifier using the LASSO Cox regression analysis. 13 genes measured at the SDM were included in the LASSO regression selection. Finally, only 4 of the 13 genes with the most significant appearances were retained as the signatures of our LASSO model (**Tables S14**, **15**), including PTPRZ1 (HR, 3.51; 95% CI, 1.76-7), SLC8A3 (HR, 4.1; 95% CI, 1.9-8.83), SIGLEC15 (HR, 0.31; 95% CI, 0.15-0.67), and CCR1 (HR, 0.12; 95% CI, 0.02-0.89) (**Figure 7D**; **Table S14**). After coefficient determination, we used the following formulas for this 4-gene model to calculate the risk score:

Risk Score = 1.486×SLC8A3+0.541×PTPRZ1−0.508×CCR1−0.552×SIGLEC15

The model’s high/low scores divided the TARGET cohort into two groups with sharply different prognoses (HR, 5.93; 95% CI, 3.01-11.68; P < 0.001). The 5-year survival rate tended to zero in the high-risk group, while it exceeded 60% in the low-risk group. This 4-gene model also separated GSE21257 patients with favorable or unfavorable prognosis based on its risk scores; high score generated statistically significant mortality (HR, 5.4; 95% CI, 2.06-14.13; P < 0.001) than the low (**Figure 7E**). Thus, the 4-gene model could act as a stable prognostic predictor across various datasets.

## 4 Discussion

This study performed transcriptome profiling and consensus-clustering analysis to classify osteosarcoma into four subtypes. Patients of S-IV were closely correlated with poor outcomes and demonstrated active cholesterol metabolism. SQLE was screened out as a potential drug target for S-IV patients within the scope of druggable genes with high-risk scores. For achieving diagnosis at initial biopsy, we developed the SDM and a 4-gene LASSO model to identify patients more likely with poor outcomes. The classification, therapeutic target, and diagnostic models were all novel to osteosarcoma and verified well by independent datasets or cellular phenotypic experiments.

Molecular classification for cancer aims to handle the drug response variability and optimize curative effects by considering underlying tumor biology (53). Previous studies concerning subtyping for osteosarcoma mainly focused on immune subgroups (17, 22). This study was the first comprehensive analysis of molecular stratification using RNA-seq data. We demonstrated integrated information on survival, immunity, and metabolic signature pathways for each subtype and proposed therapeutic recommendations for patients.

As we know, cholesterol is vital for the growth of mammalian cells (54). It is a precursor to bile acids and steroid hormones (55), which can initiate or facilitate colon, breast, and prostate cancers (56). Besides, cholesterol can also disturb signaling pathways involved in tumor growth and cancer progression (57). SQLE is a rate-limiting enzyme in cholesterol biosynthesis *via* catalyzing the first oxygenation step in sterol biosynthesis (58). This paper proposed that clinical drugs targeting SQLE could help prolong the survival time of S-IV patients. Research has reported that SQLE was markedly up-regulated (25.2-fold) in non-alcoholic fatty liver disease hepatocellular carcinoma (NAFLD-HCC) (59). Our work demonstrated that knocking down SQLE could suppress proliferating and migrating ability of osteosarcoma cells, which reinforced our belief that SQLE could play a targetable oncogenic role in osteosarcoma.

In current clinical practice, terbinafine is an anti-fungal drug used to treat cutaneous mycoses, including onychomycosis (60). It is preferably combined with topical nail lacquers such as ciclopirox and amorolfine (61). As for osteosarcoma, terbinafine has not yet been established as a clinical treatment. However, previous basic studies suggest terbinafine has broad therapeutic potential for tumors. Indeed, terbinafine inhibited angiogenesis by suppressing endothelial cell proliferation and migration (62). Additionally, combined therapies with terbinafine and nocodazole induced cell cycle arrest and apoptosis in cancer cells (63). One study regarding colorectal cancer (CRC) proved that terbinafine suppressed the growth of patient-derived organoids and inhibited the proliferation of CRC cells *in vitro* and *in vivo* (64). Apart from tumors, another study regarding nonalcoholic steatohepatitis (NASH) indicated combined terbinafine and acetazolamide synergistically ameliorated NASH in mice with superior efficacy (65).

To refine the current diagnostic regimen, we established two practical and reliable predictive models for distinguishing subtypes of patients at an early stage. Thus, patients could receive timely and efficacious drug treatment before surgery. Our models could complement existing computational frameworks relying predominately on immune infiltration or other aspects.

In addition to good prediction accuracy, the predictive models also possessed meaningful biological implications. Taking the 4-gene LASSO model as an example, previous studies have demonstrated that PTPRZ1 mediates mitotic somal translocation and glioblastoma tumor invasion (66). PTPRZ1 has been found over-expressed in various tumors such as lung cancer, cervical cancer, hepatocarcinoma, renal cancer, and glioblastoma (67). Moreover, this protein functions in cell proliferation, cell adhesion and migration, epithelial-to-mesenchymal transition, cancer stem cells, and treatment resistance by interacting with some molecules (68). It could decrease chemosensitivity in triple-negative breast cancer (TNBC) by enhancing the activation of the NF-κB signaling pathway (69). Thus, PTPRZ1 could facilitate tumor development and therefore influence patients’ prognosis. SLC8A3 was also believed to associate with a poor prognosis, but the mechanisms remained unclear (70). CCR1-chemokines were produced by osteoclasts and played an essential role in inflammatory cell chemotaxis (71). In 2018, Jennie Briard et al. found SIGLEC15 positively regulated osteoclast differentiation, and loss of it could result in impaired osteoclast differentiation and osteopetrosis in SIGLEC15-deficient mice (72).

Because of the retrospective study design, validation of classification and models in large clinical trials is needed. The putative drug targets were derived from data analysis and require further confirmation of *in vivo* experiments. Perhaps targeting cholesterol biosynthesis deserves more robust therapeutical attempts in human osteosarcoma. Despite the limitations, this study could offer a more comprehensive description and enhance our understanding of osteosarcoma. The distinct features of different subtypes delineated the complex mechanism of osteosarcoma pathogenesis. The predictive models and potential drug target SQLE might serve as valuable hints for further in-depth biological, diagnostic and therapeutic exploration.

## 5 Conclusions

In summary, stratifying osteosarcoma patients into different subtypes and establishing diagnostic models hold the possibility to enable treatment to be more precise, more individualized, and more effective than current treatments—and probably generate fewer side effects. The therapeutic target SQLE could probably be a potential key to refining traditional treatment addressing the current treatment dilemma of osteosarcoma.

## Data availability statement

The original contributions presented in the study are included in the article/**Supplementary Material**. Further inquiries can be directed to the corresponding authors.

## Author contributions

DY, AS, and FW designed the research. KZ and YH analyzed the data. YZ carried out the experiments. KZ, YH and YZ drafted the manuscript. DY, AS and FW reviewed and revised the manuscript. All authors contributed to the article and approved the submitted version.
