# Supplementary material for: Molecular features and predictive models identify the most lethal subtype and a therapeutic target for osteosarcoma
Source: Front Oncol. 2023 Feb 16;13:1111570. doi: 10.3389/fonc.2023.1111570 (PMC9980341; doi:10.3389/fonc.2023.1111570)
Supplement: Supplementary file 1 [file DataSheet_1.docx]

Supplementary Material

Molecular features and predictive models identify the most lethal subtype and a therapeutic target for osteosarcoma

Kun Zheng^1,2^^†^, Yushan Hou^1,3†^, Yiming Zhang^1,3†^, Fei Wang^2, *^, Aihua Sun^1,3*^, Dong Yang^1*^

*** Correspondence:**Dong Yang

E-mail: [yangdong@ncpsb.org.cn](mailto:yangdong@ncpsb.org.cn)

Aihua Sun

E-mail: [sunaihua@ncpsb.org.cn](mailto:sunaihua@ncpsb.org.cn)

Fei Wang,

E-mail: orthopedics.ghstc@gmail.com

^†^These authors contributed equally to this work and share first authorship.

# Supplementary Figures and Tables

## Supplementary Figures


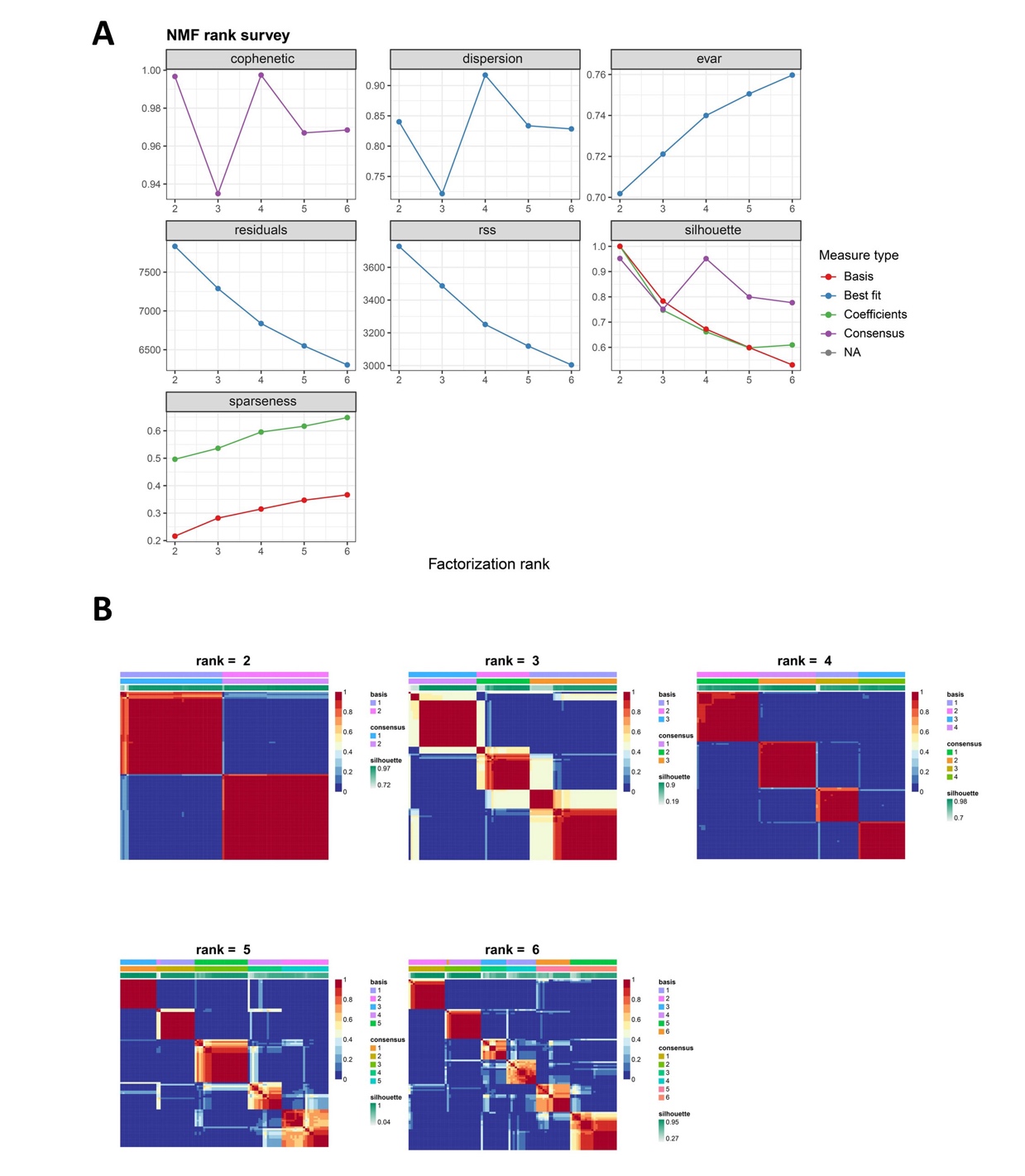


**Supplementary Figure 1.** **Nonnegative matrix factorization (NMF) classification with rank K from 2 to 6 identifies four subtypes.** (A) Estimating the rank: Quality measures were computed from 100 iterations for rank K from 2 to 6. (B) Heat map of NMF consensus matrix with rank K from 2 to 6. Based on visual inspection of the hierarchical clustering and the profiles of cophenetic correlation coefficient and average silhouette width for solutions with 2 to 6 clusters, we consider K = 4 the preferred solution.


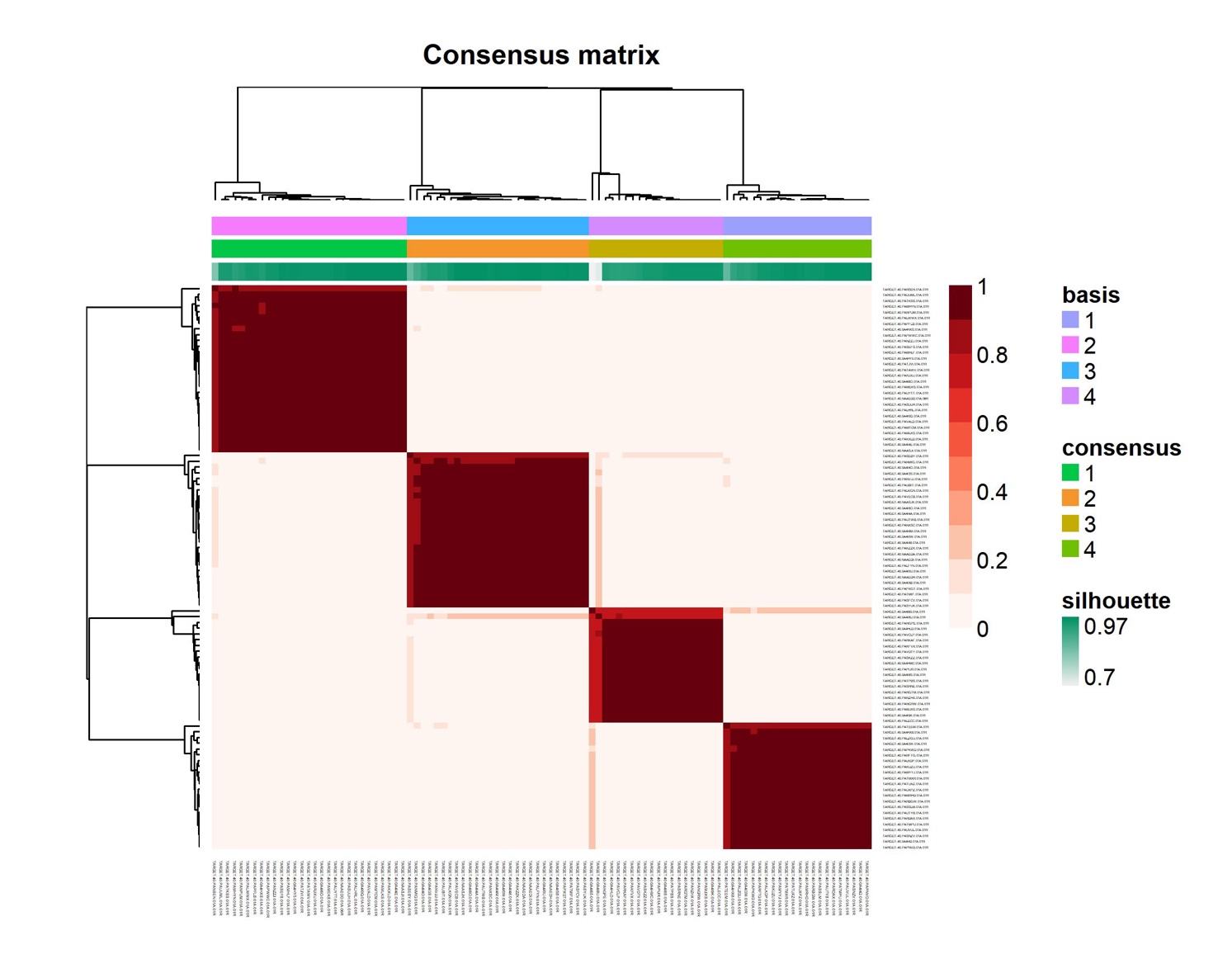


**Supplementary Figure 2.** **Consensus matrices computed from 400 runs with rank=4.**

**
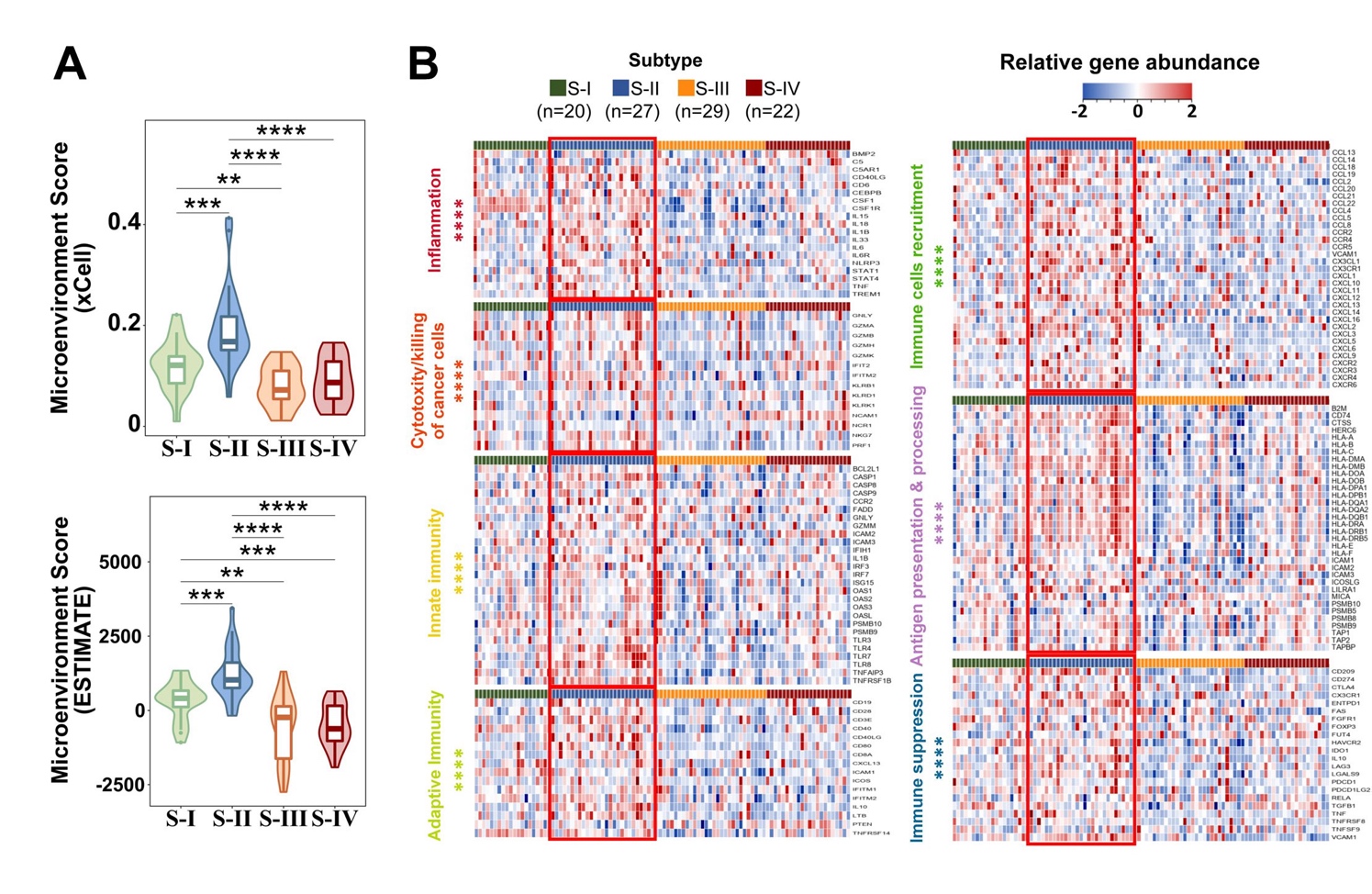
**

**Supplementary Figure 3.** **Differential immune infiltrating levels in tumors across four subtypes.** (A) Violin plots showed the distributions of the microenvironment score of each subtype computed by xCell (up) or ESTIMATE (down). The width of the violin plot represented the number of samples at the given expression level on the height. (B) Heatmap indicated the activation of various immune pathways in each subtype. S-Ⅱ showed obvious overexpression of genes involved in inflammation, cytotoxicity, innate and adaptive immunity, immune cells recruitment, antigen presentation, and immune suppression (S-Ⅱ patients *vs.* other patients). **P<0.01, ***P<0.001, ****P<0.0001. Statistical test: Wilcoxon.


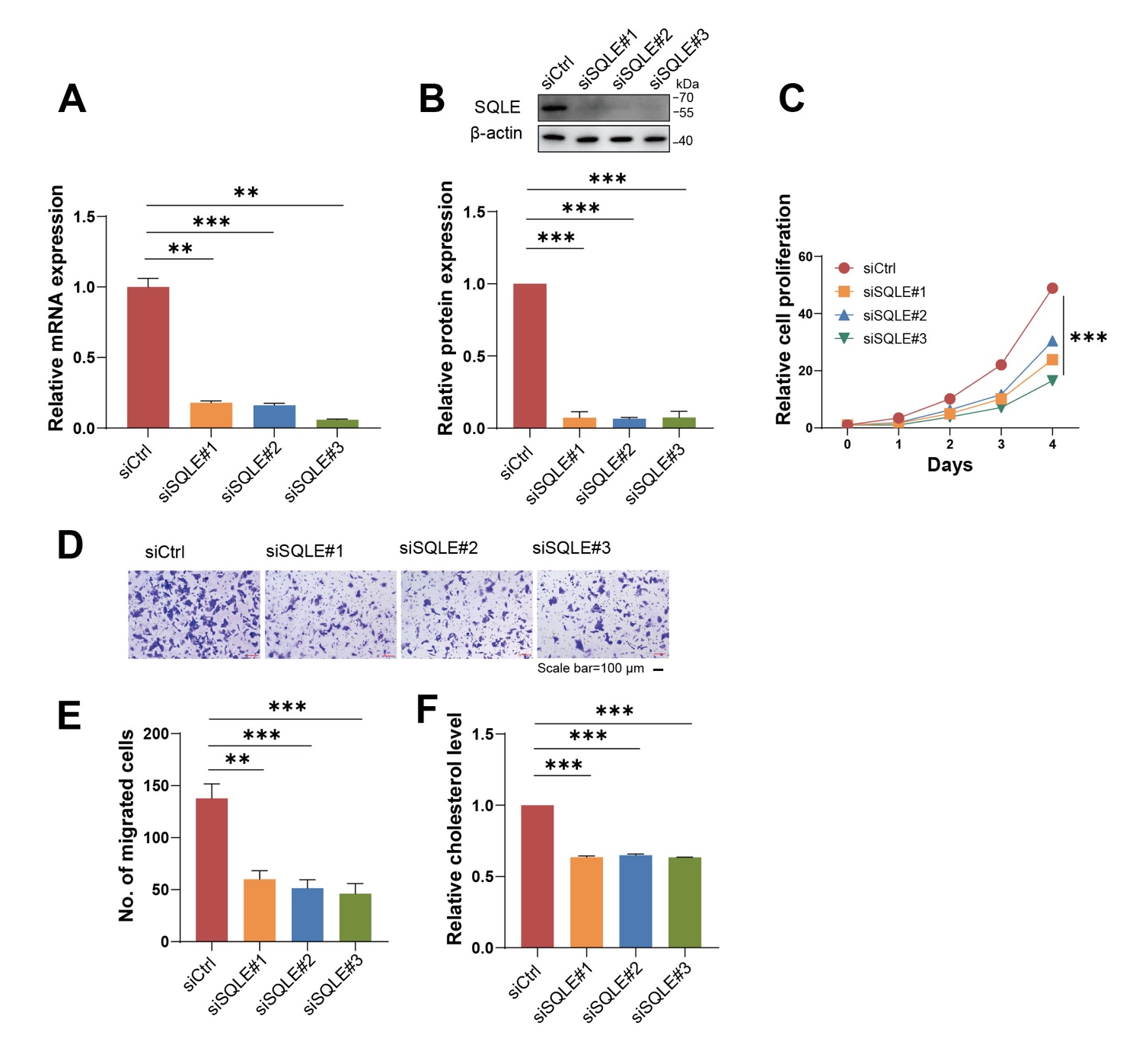


**Supplementary Figure 4. SQLE knockdown produced significant inhibition on cancer-related phenotypes of Saos-2 cells *in vitro*.** (A) Quantitative PCR (Q‑PCR) with mRNA expression confirmed the knockdown of SQLE after 24 h siRNA transfection. (B) Western blot analyses with protein expression confirmed the knockdown of SQLE after 48 h siRNA transfection. The β-actin was treated as the loading control. (C) Cell proliferation was suppressed by SQLE knockdown. (D) Cell migration was inhibited by SQLE knockdown. The scale bar represented 100 μm. (E) indicated the quantification of positive signals from (D). (F) The level of intracellular cholesterol was reduced by SQLE knockdown. Error bars represented ± SD of three biological replicates. **P < 0.01, ***P < 0.001 by Student t-test.``


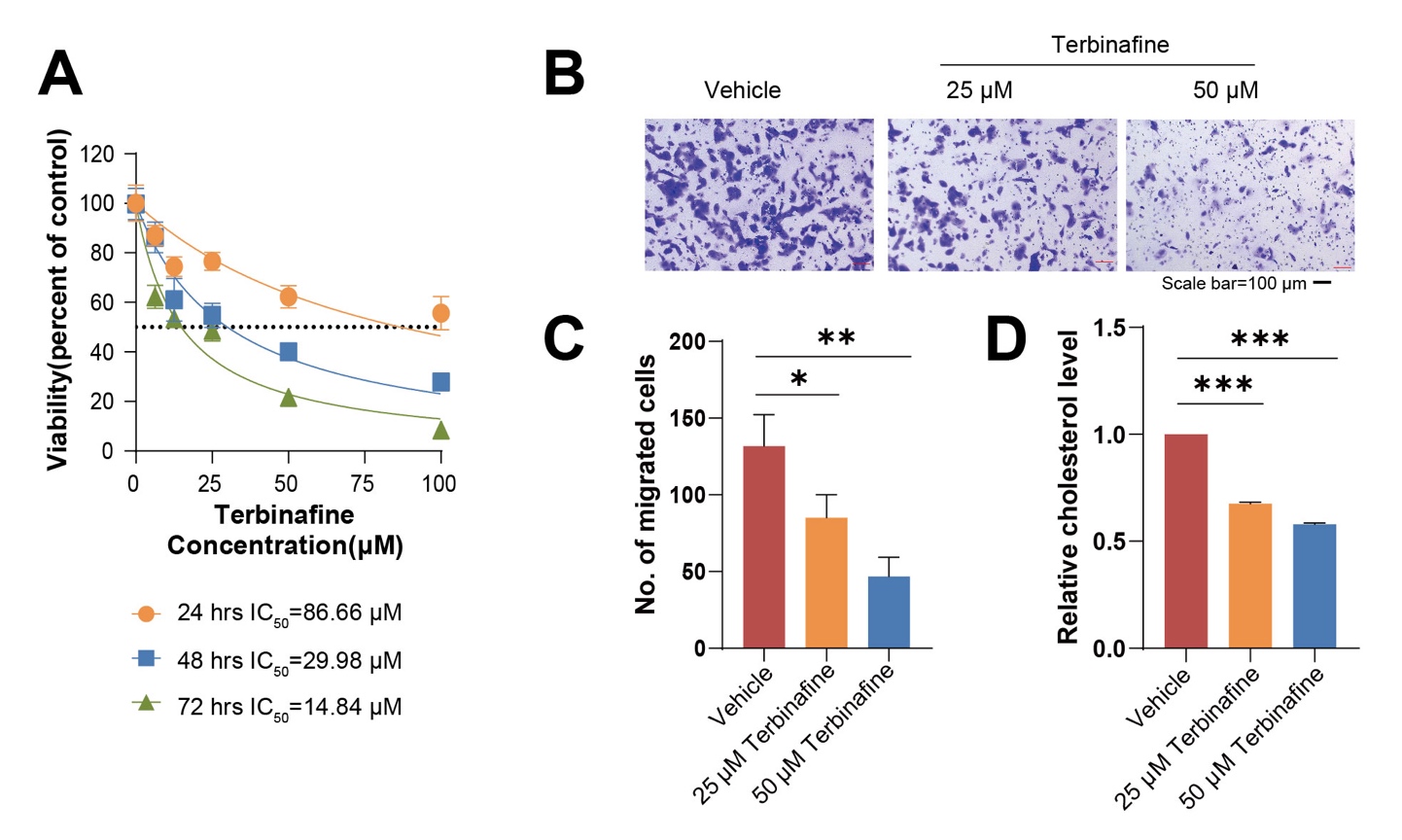


**Supplementary Figure 5.** **Terbinafine treatment impaired the growth and migration of Saos-2 cells *in vitro*.** (A) Different gradient concentrations of terbinafine were added to the Saos-2 cells. As the dosage of terbinafine increased, the viability of the cells decreased. (B) The Saos-2 cells were respectively treated with 25 μM and 50 μM terbinafine. The number of migrating cells decreased obviously with the increase in drug concentration. Scale bars, 100 μm. (C) indicated the quantification of positive signals from (B). (D) The level of intracellular cholesterol was reduced by terbinafine treatment. Error bars represent mean ± SD; statistical analysis was performed using the Student t-test. *P < 0.05, **P < 0.01, ***P < 0.001.


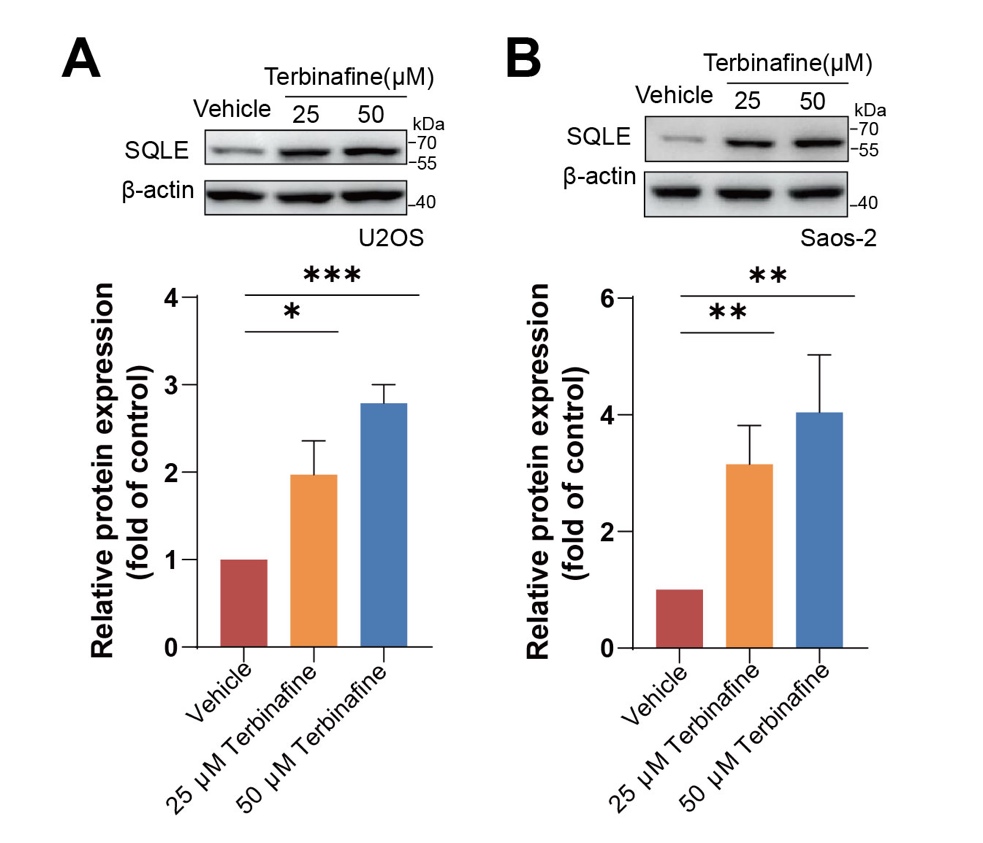


**Supplementary Figure 6. Terbinafine treatment induced the increase of SQLE** **protein levels in U2OS and Saos-2 cells.** Cells were treated with 25 μM and 50 μM terbinafine (A) The SQLE protein level in U2OS was measured by Western Blot. The β-actin was treated as the loading control. (B) The SQLE protein level in Saos-2 was measured by Western Blot. The β-actin was treated as the loading control. Error bars represent mean ± SD; statistical analysis was performed using the Student t-test. *P < 0.05, **P < 0.01, ***P < 0.001.

## Supplementary Tables

**Table S1.** Clinicopathologic information of TARGET cohort.

**Table S2.** Genomic alterations of the 87 samples in TARGET dataset based on WGS, WES, custom targeted whole gene sequencing, RNA-Seq, and SNP array analysis.

**Table S3.** Immune Score, Stroma Score and Microenvironment Score for TARGET patients.

**Table S4.** Differentially expressed genes of each type.

**Table S5.** Enriched pathways in each subtype.

**Table S6.** Expression of genes related to cholesterol metabolism in each subtype.

**Table S7.** Data description for EGA cohort.

**Table S8.** Expression levels of cholesterol biosynthesis pathway and related genes in EGA cohort.

**Table S9.** Candidates yielded by drug targets screening.

**Table S10.** Feature genes identified by PermFIT.

**Table S11.** Curated genes and performance of the subtype diagnostic model.

**Table S12.** Performance of SDM in TARGET cohort.

**Table S13.** GSE21257 dataset diagnosed by the SDM.

**Table S14.** LASSO model genes determination according to 100 iterations of 5-fold cross-validation.

**Table S15.** The frequency distribution of 13 genes occurred in minλ of LASSO regression analysis.
